# Severe Agranulocytosis following Simultaneous Administration of Chlorpromazine and Trimethoprim-Sulfamethoxazole in a Patient with Sepsis: A Possible Toxic Combination

**DOI:** 10.1155/2016/5653497

**Published:** 2016-10-18

**Authors:** Anil Jha, Hassan Ghoz, Nicholas James

**Affiliations:** ^1^Division of Internal Medicine, Steward Carney Hospital, Tufts School of Medicine, Dorchester, MA, USA; ^2^Division of Critical Care and Pulmonology, Steward Carney Hospital, Tufts School of Medicine, Dorchester, MA, USA

## Abstract

*Background*. Chlorpromazine and trimethoprim-sulfamethoxazole (TMP/SMX) are two commonly prescribed medications by physicians. Either of those medications could cause fatal drug-induced agranulocytosis, with an unclear underlying mechanism. The likelihood of simultaneous prescription of both medications is high and could hypothetically result in severe agranulocytosis that is resistant to treatment.* Case Presentation*. We are presenting a case of a patient with psychosis on chlorpromazine who was prescribed TMP/SMX for a urinary tract infection. Consequently, the patient developed severe agranulocytosis and septicemia. Patient was managed by granulocyte colony-stimulating factor; however, the time to neutrophil recovery was delayed when compared to the average reported time published by previous studies.* Conclusions*. Simultaneous use of chlorpromazine and TMP/SMX is a possible toxic combination that could induce severe agranulocytosis. Further reports are needed to confirm this observation.

## 1. Background

Chlorpromazine is a phenothiazine derivative and one of the first generation typical antipsychotics, frequently used in the management of schizophrenia [1]. Agranulocytosis is one of the rare, yet life-threating side effects of phenothiazines and particularly chlorpromazine. The risk of occurrence of agranulocytosis with chlorpromazine use is approximately 0.13 percent [2–5]. Another commonly used medication that could lead to neutropenia and agranulocytosis is the antibiotic trimethoprim-sulfamethoxazole (TMP/SMX) [6, 7]. The mechanism behind drug-induced agranulocytosis is still unclear; however the two currently proposed hypotheses are direct and immune-mediated toxicity [8]. The management of drug-induced agranulocytosis starts by discontinuing the offending medication followed by empiric antibiotic coverage and administration of granulocyte colony-stimulating factor (G-CSF) [9]. We are discussing a case of severe drug-induced agranulocytosis following the simultaneous administration of chlorpromazine and TMP/SMX in a patient presenting with sepsis, discussing possible hypotheses that could explain this observation and highlighting the response to treatment with G-CSF in such scenario.

## 2. Case Presentation

A 67-year-old Caucasian male with a history of chronic anemia, type 2 diabetes mellitus, and significant psychiatric illness was admitted to our intensive care unit (ICU) in the setting of severe hypotension. The patient had been a resident of an inpatient psychiatric facility for two months and was being treated for severe schizoaffective and bipolar disorders. His medication regimen consisted of chlorpromazine (standing dose of 500 mg oral daily, 400 mg oral at night, and 100 mg intramuscular every two hours as needed). Patient's chronic medications included spironolactone, benztropine, levothyroxine, protonix, tamsulosin, and lorazepam. His initial complete blood count (CBC) results after two months of treatment with chlorpromazine showed absolute neutrophilic count (ANC) of 5,500/*μ*L, hemoglobin (Hb) of 10.3 g/dL, and hematocrit (Hct) of 31.0%, which did not change from his baseline levels. Two weeks before his transfer to our ED, the patient was diagnosed with pan-sensitive* Escherichia coli* (*E. coli*) urinary tract infection and was started on TMP/SMX 800 mg/160 mg twice daily. The patient has no documented allergies to any antibiotics and is unknown if he was previously exposed to TMP/SMX. On the third day of TMP/SMX, his ANC dropped to 2,100 as well as his blood pressure to a lowest of 100/60 mmHg, while other blood parameters and physical examination were within normal limits. TMP/SMX was discontinued for a possible contribution to the observed neutropenia and replaced by cefpodoxime proxetil 200 mg PO twice daily for four more days. However, the patient's hypotension persisted and was eventually transferred to our ICU one week after completion of antibiotics. On arrival to the ED, the patient's blood pressure was 80/50 mm/Hg, heart rate was 105 beats/min, and he had maximum temperature of 101.7 F. His CBC showed a drop in his ANC to the 30/*μ*L, Hb 6.4 g/dL, Hct 19.9%, and platelets of 200,000/*μ*L. Patient met the criteria for septic shock and work-up for sepsis revealed* E. coli* bacteremia. His CT scan showed a dilated right kidney with significant perinephric fat stranding and dilatation of the renal pelvis and proximal ureter suggesting pyelonephritis. His chest X-ray showed no infiltrates. Extensive hematologic work-up including levels of haptoglobin, lactate dehydrogenase, reticulocytic count, blood smears, and flow cytometry was within normal limits. Viral serology for cytomegalovirus, Epstein-Barr virus, parvovirus B19, and HIV were negative.

**Figure 1:**
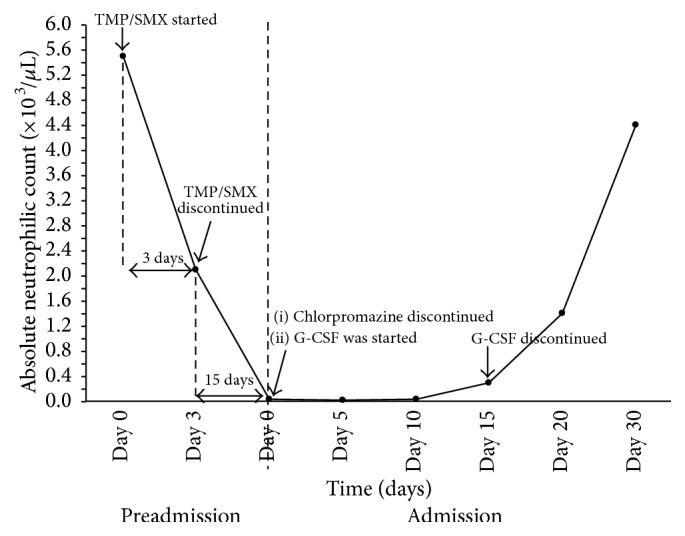
Timeline of absolute neutrophilic count changes.

On the day of admission, chlorpromazine was held, the patient was started on cefepime and vancomycin for empiric coverage of infection, and granulocyte colony-stimulating factor (G-CSF) was given at the recommended dose of 5 mcg/kg per day for 15 days. On day four, the patient's blood pressure normalized, repeat blood cultures were negative, and Hb and Hct levels responded well to blood transfusion. However, the ANC showed a slow, gradual improvement to reach 340/*μ*L after 15 days from admission and reached its baseline of 4,400/*μ*L after 4 weeks (Figure 1).

## 3. Discussion

Chlorpromazine is a frequently prescribed medication for treatment of various psychotic disorders [1]. Induced agranulocytosis is a rare, yet life-threatening adverse effect of chlorpromazine [10, 11]. One of the earliest case studies reporting chlorpromazine-induced agranulocytosis was published in 1954 [12], following which several case reports were published describing the same clinical observation [2, 13, 14]. TMP/SMX has also been reported to cause agranulocytosis [15]. The mechanism behind the hemotoxic effects of both drugs is still unclear. Studies suggest that chlorpromazine can indirectly affect cell division by inhibiting enzymes involved in cellular DNA synthesis such as DNA polymerase, thymidylate kinase, and RNA polymerase [16]. Data regarding sulfamethoxazole toxic effects on white blood cells is limited and suggests it to be rare, idiosyncratic, and dose-dependent toxicity [17]. While TMP/SMX induced agranulocytosis and neutropenia usually require previous sensitization such as previous exposure and hypersensitivity reactions, neutropenia following an acute ingestion is unique but predictable in view of TMP/SMX toxic effects on white cells. In 2003, Andrès et al. published prospective data that involved 9 cases of TMP/SMX induced agranulocytosis. The range of doses of TMP/SMX in those cases was from 800 to 2400 mg/day, the duration of drug intake ranged from 3 to 17 days, median age of patients was 69 years, male-female ratio was 2, and the main clinical presentation included septicemia and septic shock [18]. Although our patient possesses similar characteristics as those nine cases, the presence of chlorpromazine in our case adds other possible hypotheses for the observed neutropenia besides TMP/SMX induced agranulocytosis. For example, the observed neutropenia could be related to chlorpromazine-induced bone marrow toxicity that reduced neutropoiesis and subsequently reached the peripheral blood and presented as agranulocytosis. Our proposed hypothesis combines the aforementioned possibilities concluding that TMP/SMX and chlorpromazine have both contributed to the observed agranulocytosis and was based on the following: (i) presence of risk factors for toxic agranulocytosis that includes old age, association of chlorpromazine with other drugs known to be able to induce agranulocytosis, and past history of use of high doses of chlorpromazine; (ii) normal ANC despite two months of continuous chlorpromazine use; (iii) sudden severe drop in ANC following high dose TMP/SMX administration; (iv) ANC which did not improve after 1 week of discontinuing TMP/SMX and required the discontinuation of chlorpromazine as well.

The initial management of drug-induced agranulocytosis is to discontinue the offending medication. Studies have shown controversial benefit from using granulocyte colony-stimulating factor (G-CSF) in the management of drug-induced agranulocytosis; however, the majority described shorter recovery periods, especially if in the setting of sepsis [9, 19]. A systematic review by Andersohn et al. has shown that the time to neutrophil recovery after discontinuing either sulfamethoxazole or chlorpromazine (average use of 42–45 days) was around eleven days without treatment with G-CSF. Sepsis was not an exclusion criterion in that study [20].

In our case, after treatment with discontinuation of both drugs and initiating G-CSF, the patient's neutrophil count fully recovered after almost 4 weeks (Figure 1). This is almost double the time when compared to previously published data of either of the medications separately (eleven days) [20]. Thus, we hypothesize that the use of TMP/SMX in a patient on chlorpromazine could result in long lasting, severe agranulocytosis.

This case report highlights the possible risk of concomitant exposure to two commonly prescribed medications, TMP/SMX and chlorpromazine, in inducing agranulocytosis and brings attention to physicians to be cautions when simultaneously prescribing these medications. More cases need to be reported to confirm such finding.

## Abbreviations

WHO: World Health Organization
G-CSF: Granulocyte colony-stimulating factors
TMP/SMX: Trimethoprim-sulfamethoxazole
ANC: Absolute neutrophilic count
WBC: White blood cells count.
